# An Investigation into Video Game Addiction in Pre-Adolescents and Adolescents: A Cross-Sectional Study

**DOI:** 10.3390/medicina56050221

**Published:** 2020-05-06

**Authors:** Maria Rosaria Esposito, Nicola Serra, Assunta Guillari, Silvio Simeone, Franca Sarracino, Grazia Isabella Continisio, Teresa Rea

**Affiliations:** 1 Istituto Nazionale Tumori “Fondazione G. Pascale”, via M. Semmola, 80131 Naples, Italy; m.rosiespo@gmail.com; 2 Department of Molecular Medicine and Medical Biotechnology, University Federico II of Naples, via S. Pansini, 80131 Naples, Italy; 3 Department of Public Health, University Federico II of Naples, via S. Pansini, 80131 Naples, Italy; aguillari70@gmail.com (A.G.); teresa.rea@unina.it (T.R.); 4 Department of Biomedicine and Prevention, University of Rome “Tor Vergata,” Montpellier 1, 00133 Rome, Italy; silviocecilia@libero.it; 5 Department of Pediatrics, Betania Evangelical Hospital, via Argine, 80147 Naples, Italy; francasarracino@tiscali.it; 6 Continuing Medical Education Unit, University Federico II of Naples, via S. Pansini, 80131 Naples, Italy; continis@unina.it

**Keywords:** video game addiction, pre-adolescents and adolescents, gaming disorder, Game Addiction Scale (GAS)

## Abstract

*Background and Objectives*: Game addiction is an emerging problem in public health. A gaming disorder is characterized by a pattern of persistent or recurrent gaming behavior. The behavioral pattern is severe enough to implicate a significant involvement of family, social, educational, professional, or other relationships. Therefore, greater attention needs to be paid to potential addictive behaviors in terms of video games in order to identify both pre-adolescents and adolescents at risk and to provide them with adequate assistance. *Materials and Methods*: A random sample of 622 students including pre-adolescents and adolescents were enrolled from September 1st to October 31th 2016, and the Game Addiction Scale (GAS) interview was used to identify pathological students with both Monothetic and Polythetic analysis. *Results*: This study shows the presence of pathological students is equal to 1.93%, with 37.46% and 4.50% obtained with Monothetic and Polythetic analysis (global and partial), respectively. In our sample, the most frequent were students with a gaming time of 1 or 2 h, and students with a day gaming frequency of 1, 2, or 3 times a day. The items with more pathological students were Item 2 (i.e., Tolerance) and 4 (i.e., Withdrawal). Every item was positively correlated with Daily gaming time (hours) and Daily game frequency, excluding Item 4 (i.e., Withdrawal). Finally, the Monothetic GAS score was positively correlated with Daily gaming time while the Polythetic Global GAS was positively correlated with Daily game frequency and negatively with Education level; instead, the Polythetic Partial GAS score was positively correlated with only Daily gaming time. *Conclusion*: Males are pathological gamblers more so than females and spend more time playing video games. An increase in Daily game frequency or Daily gaming time implicates an increase in video game addictions, while an increase in Education level, which generally corresponds to a greater age, implicates a decrease in game addiction. Finally, we observed that the correlations obtained between the Polythetic Partial GAS score with the independent variables such as Age, Gender, Education level, Daily gaming time (hours), and Daily game frequency were analogous to the significant correlations obtained with the Monothetic GAS score, while these correlations were different for the Polythetic Global GAS and the independent variables. These results suggest that the use of the original Polythetic scale should not be neglected.

## 1. Introduction

Most of the free time dedicated to recreational activities is represented by “video games,” which respond to a “playful need,” captivating people of all ages, sexes, and social classes [1]. The video game industry is prevalent worldwide, with products running on mobile phones, computers, and video game devices, showing positive effects on basic mental processes such as perception, attention, memory, and decision-making [2]. However, abuse of such a play activity can become an addiction, i.e., a video game addiction [3].

The World Health Organization (WHO) included gaming disorder, both online (Internet computerized games) and offline (non-Internet computerized games), for the first time in the draft of 11th revision of the International Classification of Diseases (ICD-11). This problem has also been confirmed in the 5th edition of the Diagnostic and Statistical Manual of Mental Disorders (DSM-5), which includes diagnostic criteria for Internet gaming disorders [4].

Many studies have examined Internet use problems, including online gaming, while other authors have focused their attention only on problems connected to online gaming, such as interpersonal relations and school/social functions [5,6], impulsivity and neuroticism [7], aggression and violence [8], attention deficit [9], depression [10,11], anxiety [10,12], sleep problems (including subjective insomnia and poor sleep quality [13]), and spending money on gaming [6].

A systematic review [1] on Internet gambling disorder (IGD) reported a higher prevalence in males than in females and among young people compared to the elderly [6]. Variations were also observed based on the geographical position [6,7,9,11,14,15,16,17]; For example, in Italy [18], the prevalence among adolescents is about 9%, which is comparable to that of the main European nations.

Gaming disorders were initially identified as having the characteristics, normally evident over a period of at least 12 months, of a pattern of persistent or recurrent gaming behavior (i.e., inability to control excessive gaming) [19], which may be in relation to online (i.e., over the internet) or offline games [20,21], and which severely impacts on the family, social, educational, professional, or other relationships of the player.

In addition, there is a need for studies with greater heterogeneity to understand potentially addictive behavior, including that related to video games [22]. In fact, there is a disagreement between some authors on the inclusion of the term disturbance of the game, stemmed from the fact that gambling may not be the most appropriate starting point for considering behavioral addiction [23]. However, there is still an open debate to identify addiction syndrome in comparison to behavioral dependence in a more specific way in order not to pathologize common behaviors [24]. Furthermore, it is not yet clear how behaviors considered pathological can be related to the duration of the game, or how to understand the seriousness of dependent behaviors [25]. This association is due to the fact that a gaming disorder shares many features with addictions related to psychoactive substances and with gambling disorder [26]. The American Psychiatric Association applies to this behavior the same diagnostic criteria used for substance addictions, to new forms of behavioral addiction [4]. Behavioral addictions—and therefore, those including video games—according to some experts, should be placed among “Impulse Behavior Disorders,” as is already the case for gambling [27].

A variety of terms have been employed to describe this condition including: Excessive Internet addiction (encompassing gaming, social networking sites, and video viewing) or video game addiction; problematic computer game use [28], problematic video gaming [29], video game dependency, or pathological video gaming [7,30]; or excessive gaming, pathological gaming, video game addiction, digital game addiction, or online gaming addiction [26,27].

These behaviors are measured in various subjects (e.g., children, adolescents, gamers) and geographical areas [31]. In this study, video game addiction is used as the preferred term to refer to the problematic or pathological use of video games. Lemmens et al. defined video game addiction as an “excessive and compulsive use of computer or video games that results in social and/or emotional problems; despite these problems, the gamer is unable to control this excessive use” [3]. Moreover, the time spent playing video games, and specifically a higher frequency and gaming time, are strongly correlated with symptoms of pathological gaming [19,32,33,34].

These signs of addiction are adapted to adolescent video game playing as follows: [35] Increased time spent playing or thinking about video games, scheduling the next game or remembering previous games, bad mood or irritability when it is not possible to play, increased time spent playing during difficult times, failed attempts to control game playing time, concealing the time spent playing from parents or friends, failing to do homework, lying about it in order to play video games, losing hours of sleep, missing meals, and spending less time with family or friends in order to play more. There is greater agreement that gaming problems are connected to other negative factors. Some studies claim that Internet or video games addictions are associated with psychiatric co-morbidities [36], such as depression [30], substance abuse (e.g., alcohol [37]), attention deficit disorder [38], decreased academic achievement [39], and problematic conduct [32]. In particular Brunborg et al., 2014, found that youths who had problematic conduct or were addicted gamers had a greater risk of feeling low, irritable, or nervous, of being in a bad mood, tired, or exhausted, and of feeling afraid when compared to non-problem gamers [32].

Unfortunately, proving a prevalence rate for video games addiction is not easy because a variety of assessment tools have been used in related studies, as well as different population types and classifications or cutoffs for problematic, addicted, or pathologic gamers.

For this study, we used the scale developed by Lemmens et al. to assess gaming among adolescents [3]; this tool has already been used by other authors [15,35,40,41], and is conceptually based on the criteria for pathological gambling, described by the fourth edition of the DSM.

In our study, we focused on pre-adolescents and adolescents because they generally play computer and video games more frequently than adults [42] and are considered more vulnerable to gambling addiction than adults [40].

The objective of this study was to investigate video game addiction and the associated risk factors using a sample of pre-adolescent and adolescent students in Southern Italy.

## 2. Materials and Methods

### 2.1. Study Design and Population

A cross-sectional study was conducted from September 1st to October 31th 2016 using a sample of 622 pre-adolescent and adolescent students recruited from two primary (*n* = 217) and two secondary (*n* = 405) schools.

Informed consent was signed by all parents of the students included in this study. For all students anonymity was guaranteed. No economic incentives were offered or provided for participation in this study. This study was approved by “Circolo Didattico Giancarlo Siani, Mugnano (Naples), prot. 2366 (May 19, 2015),” “Circolo Didattico Giovanni Falcone, Melito (Naples), prot. 1113/B32 (May 20, 2015),” “Scuola Secondaria Statale di I Grado, Marino Guarano, Melito (Naples), prot. 1279/C23 (May 25, 2015),” and “Scuola Secondaria Statale di I Grado, Illuminato-Cirino, Mugnano (Naples), prot. 3240/C12 (September 30, 2015).”

All teachers involved in this study were informed about the purpose and method of investigation and were trained by the research team. All information and data were archived according to Legislative Decree 196/2003. The study was performed in accordance with the ethical considerations of the Helsinki Declaration. 

The questionnaire, based on the Game Addiction Scale (GAS), was administered to students after a brief explanation. Some teachers were present in the classroom during the completion of the questionnaire, to help the students with its completion. All students aged under 10 years were excluded from the study.

### 2.2. Instrument

The questionnaire was structured in two parts; the first part was designed to collect information about variables including *Age*, *Gender*, *Education level*, *Daily gaming time*, and *Daily game frequency*.

The second part was based on the Game Addiction Scale (GAS) [3], i.e., a tool developed to measure computer and video game addiction among adolescents using the clinical criteria for pathological gambling in the DSM-5 [43].

The short version of the GAS includes seven Items: S*alience* (Item 1): Playing a game becomes the most important activity in a person’s life and dominates his thinking (preoccupation), feelings (cravings), and behavior; *Tolerance* (Item 2): The process where a person begins playing games more often, gradually building up the amount of time spent on games; *Mood modification* (Item 3): The subjective experiences that people report as a result of engagement in games; *Withdrawal* (Item 4): Unpleasant emotions and/or physical effects that occur when game play is suddenly reduced or discontinued, i.e., it consists mostly of moodiness and irritability, but may also include physiological symptoms, such as shaking; *Relapse* (Item 5): The tendency to repeatedly revert to earlier patterns of game play—excessive playing patterns are quickly restored after periods of abstinence or control; *Conflict* (Item 6): This refers to all interpersonal conflicts resulting from excessive gaming—conflicts exist between the player and those around him, and may include arguments and neglect, but also lies and deception; *Problems* (Item 7): This refers to problems caused by excessive gaming time—it mainly concerns displacement problems as the object of addiction takes preference over activities, such as school, work, and socializing.

Each item of the GAS is preceded by the statement “During the last six months, how often…” and is scored with a 5-point Likert scale (1 = never, 2 = rarely, 3 = sometimes, 4 = often, and 5 = very often). Lemmens et al. suggested two forms for evaluation of the presence of game addiction: One *Monothetic form* and two possible polythetic forms [3]. *Monothetic form* refers to when the pathologic subject is identified only by all items having a score ≥ 3; *Polythetic form 1* (*Polythetic Global GAS*) refers to when the pathologic subject is identified only by at least half of the items having a score ≥ 3; and *Polythetic form 2 (Polythetic partial GAS*) refers to when the pathologic subject is identified only by Items 4, 5, 6, and 7 simultaneously having a score ≥ 3.

### 2.3. Cultural Validation of the Gas and Preliminary Testing

The translation and cultural adaptation of the GAS tool has followed the phases of the model proposed by the World Health Organization [44]. With the authors’ help, as well as that of bilingual and non-bilingual translators, and of the support of experts, the Italian version of the GAS tool was defined. The aim of the translation and validation process was to produce a new language version of the GAS tool that was conceptually equivalent to the original version. The correct validation process of this model led to an equivalent tool that can be used in a new cultural reality, and it represents a correct conceptual translation rather than a simple literary translation. In order to test the scale, a pilot study was carried out on a random sample of 15 students (9 pre-adolescents and 6 adolescents with 10 males and 5 females) to make sure the respondents were interpreting the questions as intended, prior to carrying out the study on a larger scale. The questions were determined to be clear and understandable, as the children had no difficulty in answering them.

### 2.4. Statistical Analysis

Statistical analysis was performed using the Matrix Laboratory (MATLAB) Statistical toolbox version 2008 (MathWorks, Natick, MA, USA). Data are presented as numbers and percentages for categorical variables, and continuous data expressed as the mean ± standard deviation (SD) unless otherwise specified. A binomial test was performed to compare two mutually exclusive proportions. A multiple comparison chi-square test was used to define significant differences among percentages. In this case, if the chi-square test was significant (*p* < 0.05), a post hoc Z-test was performed. In the case of paired data, a multiple comparison Cochran’s Q test was used to compare the differences among percentages under the consideration of the null hypothesis that there were no differences between the variables. When the Cochran’s Q test was positive (*p* < 0.05), then a minimum required difference for a significant difference between two proportions was calculated using the Minimum Required Differences method with Bonferroni *p*-value corrected for multiple comparisons according to Sheskin [45]. Finally, to perform both linear and logistic regression, we considered the following variables: *Item n* (*n* = 1, ..., 7): pathologic = 1 (score ≥ 3); non-pathologic = 0 (score < 3); *Monothetic GAS score*: pathologic = 1 (all items with a score ≥ 3); non-pathologic = 0; *Polythetic Global GAS score*: pathologic = 1 (subjects with a minimum of 4 out of 7 Items with a score ≥ 3); non-pathologic = 0; *Polythetic Partial GAS score*: pathologic = 1 (subjects with Items 4, 5, 6 and 7 simultaneously having a score ≥ 3); non-pathologic = 0; *Gender*: Male = 0 and female = 1; *Education level*: elementary school degree = 1, middle school degree = 2; *Daily gaming time*: 1 h = 1, 2 h = 2, 3 h = 3, 4 h = 4, greater or equal to 5 h = 5; *Daily game frequency*: one = 1, two = 2, three = 3, greater or equal to four = 4.

Univariate and multivariate linear correlation analyses were also performed. In this case the test on Pearson’s linear correlation coefficient *R* was performed with Student’s *t*-test, under the null hypothesis of Pearson’s linear correlation coefficient *R* = 0. For this we considered the dependent variable as every Item (Item:1=*Salience*, 2=*Tolerance*, 3=*Mood modification*, 4=*Withdrawal*; 5=*Relapse*, 6=*Conflict*, 7=*Problem*), using a 5-point Likert scale, and the independent variables as: *Age*, *Gender*, *Education level*, *Daily gaming time*, and *Daily game frequency*. Instead, logistic regression was performed to analyze the relationship between the *Monothetic GAS score, Polythetic Global GAS score,* and *Polythetic Partial GAS score* with independent variables: *Age*, *Gender*, *Education level*, *Daily gaming time*, and *Daily game frequency*. All tests with *p* < 0.05 were considered significant.

## 3. Results

The student sample was composed of 52.89% males and 47.11% females, all of Italian ethnicity, with an age range 10–15 years old (a mean of 11.5 years old and a standard deviation equal to 1.28 years old), as shown in Table 1. In particular Table 1 shows the characteristics of the sample in our study, with interviews and statistical analysis performed among the modalities for every variable.

The results show a significant presence of students who attended lower secondary school in comparison to elementary school (65.11% > 34.89%, *p* < 0.0001).

Regarding *Day gaming time*, the significantly most frequent answers were two (32.15%) and three hours (34.24%), while one (12.38%), four (12.54%), and greater or equal to five hours (1.29%) were the significantly less frequent answers. In regard to *Day gaming frequency*, the most frequent answers were one (32.15%), two(34.24%), and three (32.48%), while greater or equal to four was given significantly less frequent(0.96%). In addition, many students were pathological in terms of Item 2 (64.15%, *p* < 0.0001) and Item 4 (60.13%, *p* < 0.0001), while few students were pathological in terms of Item 3 (20.26%, *p* < 0.0001) and Item 7 (23.47%, *p* < 0.0001). Finally, according to GAS classification [3], we observed a low presence of pathological students, i.e., 1.93% (12/622), by *Monothetic GAS score*. A similar case was also seen for *Polythetic Partial GAS score*—4.50% (22/622)—while for *Polythetic Global GAS score,* more students were at risk, i.e., 37.46% (233/622).

In Table 2, the percentages of the students’answers for every item and every score defined by a Likert scale of 1–5 are shown.

Table 3 shows the univariate and multivariate linear correlation analyses, considering the independent variables as *Age*, *Gender*, *Education level*, *Daily gaming time,* and *Daily game frequency*, and the dependent variable as every item considered singularly.

As can be seen, more significant correlations were individuated. Particularly for Item 1 (i.e., *Salience*), the univariate analysis showed a low but significant negative correlation with *Age*, *Gender*, and *Education level*; in other words, an increase (decrease) in *Age,* in the presence of females, and in students with a greater *Education level* implicate a decrease(increase) in the Item 1 score. In contrast, *Daily gaming time* and *Daily game frequency* were positively correlated with Item 1, i.e., an increase (decrease) in *Daily gaming time* and *Daily game frequency* implicate an increase (decrease) in the Item 1 score. In the multivariate analysis, *Daily gaming time* and *Daily game frequency* were positive predictors in comparison to the others for Item 1, while *Education level* and *Gender*, were negative predictors, confirming the results of the univariate analysis.

For Item 2, in the univariate analysis, analogous results for Item 1 were observed, apart from *Education level*, which was not correlated with Item 2. In the multivariate analysis, the positive and negative predictors were *Daily gaming time* and *Gender* respectively, in comparison to the other variables, i.e., an increase (decrease) in *Daily gaming time* and in the presence of males were predictors of an increase (decrease) of Item 2 score (i.e., *Tolerance*).

In the univariate analysis, Item 3 (i.e., *Mood modification*) was positively correlated with *Daily gaming time* and *Daily game frequency*, i.e., mood swings increase (decrease) with an increase (decrease) in *Daily gaming time* and/or *Daily game frequency*. In the multivariate analysis, only *Daily gaming time* represented a significant positive predictor for mood swings in comparison to the other variables, i.e., the presence of greater *Daily gaming time* implicates greater mood swings.

For Item 4 (i.e., *Withdrawal*), no significant correlations in either the univariate or the multivariate analysis were observed. In other words, among *Age*, *Gender*, *Education level*, *Daily gaming time*, and *Daily gaming frequency*, none had a significant impact on students, in terms of unpleasant emotions and/or physical effects, when the game was suddenly reduced or suspended.

For Item 5 (i.e., *Relapse*) in the univariate analysis, the results were analogous to those for Item 1. In other words, only *Daily gaming time*, *Daily game frequency,* and the presence of males induced the tendency to return several times to previous models of video game use, while conversely, an increase in *Age* or *Education level* implicates a reduction of this tendency. In the multivariate analysis, *Daily gaming time* and *Daily game frequency* represented two significant positive predictors for *Relapse* in comparison to the others.

In the univariate analysis, Item 6 (i.e., *Conflict*) was positively correlated with *Daily gaming time* and *Daily game frequency*, i.e., the interpersonal conflicts resulting from excessive game play increased(decreased) with an increase (decrease) in *Daily gaming time* and/or *Daily game frequency*. In the multivariate analysis only *Daily gaming time* represented a significant positive predictor for interpersonal conflicts in comparison to the others.

Finally, in the univariate analysis, we observed for Item 7 (i.e., *Problems*), analogous results to those for Item 1. Specifically an increase in *Daily gaming time, Daily game frequency*, and the presence of males was connected to problems caused by excessive video game use such as reduction of school-related activities, work, and socialization. In comparison, *Age* and *Education level* implicate a reduction of these problems. In the multivariate analysis, we observed that only *Age, Daily gaming time*, and *Daily game frequency* were significant positive predictors for Item 7, i.e., students with a greater *Age*, and an increase in *Daily gaming time* and *Daily gaming frequency* were more exposed to problems caused by excessive video game use.

In Table 4, we reported the results of the logistic regression between the *Monothetic GAS*, *Polythetic Global GAS*, and *Polythetic Partial GAS* scores and the independent variables (i.e., *Age*, *Gender*, *Education level*, *Daily gaming time*, and *Daily game frequency*).

For this scope, two models were considered. The null model: −2ln(L_0_), where L_0_ was the likelihood of obtaining the observations if the independent variables did not affect the outcome, and the full model: −2ln(L_0_), where L_0_ was the likelihood of obtaining the observations with all independent variables incorporated in the model. The difference between these two yields was estimated with the chi-square test, to define how well the independent variables affect the outcome or dependent variable; if chi-square test was positive (*p* < 0.05), than there was evidence that at least one of the independent variables contributes to the prediction of the outcome.

By using logistic regression, it was shown that *Daily gaming time* was positively correlated with *Monothetic GAS score* (Odds Ratio (OR) = 3.70 and *p* = 0.0057); in other words, an increase (decrease) in *Daily gaming time* contributes to an increase (decrease) in the *Monothetic GAS score*, i.e., to pathologic (non-pathologic) comportment in students in comparison to the other variables.

Regarding the *Polythetic Global GAS score*, the variables *Daily gaming time* and *Daily game frequency* were significantly positively correlated with it (OR = 2.64 and *p* < 0.0001, OR = 1.40 and *p* = 0.0063, respectively); in other words, an increase (decrease) in *Daily gaming time* or *Daily game frequency* contributes to an increase (decrease) in the *Polythetic Global GAS score*, i.e., individualized pathologic (non-pathologic) comportment in students in comparison to the other variables. Finally, the variable *Daily gaming time* was positively correlated with *Polythetic Partial GAS score* (OR = 2.16 and *p* = 0.0059); in other words, an increase (decrease) in *Daily gaming time* contributes to an increase (decrease) in the *Polythetic Partial GAS score*, i.e., individualized pathologic (non-pathologic) comportment in students.

## 4. Discussion

In this study we investigated the dependence on video games in adolescent and pre-adolescent students in Naples province, considering every item of the Game Addiction Scale (GAS) and factors such as *Age*, *Gender*, *Education level, Daily gaming time*, and *Daily gaming frequency*.

For this scope, we considered both *Monothetic* and *Polythetic* GAS structure. By *Monothetic* structure, i.e., considering students with all item scores greater or equal to 3 as pathologic, it was shown that only 1.93% of students were classified as problem gamblers, which is less than in other studies on pathological players, such as in that by Khazaal, Chatton et al. 2016, where it was reported that 2.3% of respondents were classified as problem gamblers, using Monothetic scale [15]. However, this is a percentage that underestimates pathological players according to other authors [46,47]; meanwhile, when using the *Polythetic* structure, we observed in our study that 37.46% of the students were classified as problem gamblers. Analogous results were obtained in France with 156 and 306 adolescents, where the authors declared that the *Monothetic* structure could underestimate pathological gamblers; in fact, only 0.6% and 1% of the adolescents, respectively, were classified as problematic video game players, while 28% were identified as excessive pathological players when using the *Polythetic* structure [35]. Instead, Wang et al., using a sample of 503 students in middle secondary school in Hong Kong, reported a prevalence of 15.6% of players who could be considered problematic [6]. However, our results show different outcomes and this could be due to various reasons. First of all, the GAS score was viewed as the *Polythetic score* by the authors, because in the DSM-5 the symptoms of internet gaming disorder should have been present for at least three months, while Lemmens et al. suggested that all seven items must occur at least sometimes in the last six months in order to indicate video game addiction [3]. However, this approach could underestimate the results of pathological gaming reported in the study of Wang et al. [6].

Secondly, in our study, both online and offline games were considered, which contributes to a greater inclusion of video games available for children with consoles and CD ROMs or DVD games [22].

Thirdly, unlike the study carried out in Hong Kong by Wang et al. [6] where they considered only secondary school students (age > 11 years), we considered students aged 10 years and above. In a longitudinal study carried out in the primary and secondary schools of Singapore, using a population of 3034 children aged 8 years and above, although in the youngest age group, only 1% of children became a pathological player it seems that the pathological game is not simply a “phase” that passes in most children [30].

Our study showed different GAS scores, in comparison to studies performed in Norway [31,32], where the authors considered an additional subdivision of the players, dividing them into dependent, problematic, and very committed by those who are not dependent.

Generally, in the literature, all items of the GAS are used to identify both pathological and non-pathological players, but some authors had affirmed that some items of the GAS could be considered as marginal criteria, such as *Salience*, *Tolerance*, and *Mood modification*, while items such as *Withdrawal*, *Relapse*, *Conflict*, and *Problems* can be considered fundamental in comparison to others [46].

In this study, we considered, in addition, the classification of the items of the GAS suggested by Fergusson Coulson et al. [46]; therefore, we identified 4.5% as problematic students (*Polythetic Partial* GAS score). This result is higher than the 1.2% and 0.89% of the two Norwegian studies, performed using samples of 10,081 subjects with an age range of 16–74 years old and of 3389 subjects an age range also of 16–74 years old, respectively [31,41], while our *Monothetic GAS* score results were similar to their 0.7% [41] and 1.41%, respectively [31].

It is likely that our sample is comparable to the stratified sample in the study of Wittek et al, where the authors observed that immigrants in Norway such as African, Asian, and both South and Middle American populations were positively associated with addicted and problem gamers, in contrast to those born in Norway [31]. In fact Wittek et al. affirmed that these immigrants are more susceptible to developing video game addiction, similarly to our Italian population, due to their rooted interest in gaming.

The regression analysis identified more significant correlations. In particular considering every item, we observed that age was negatively correlated with *Salience* and *Relapse* only in the univariate analysis, and positively correlated with *Problems* both in the univariate and the multivariate analyses. Instead, for the variable *Gender*, a negative correlation was observed with *Salience, Tolerance*, and *Relapse*, both in the univariate and the multivariate analyses; meanwhile a negative correlation only in the univariate analysis with *Problems* and a positive correlation only in the multivariate analysis with *Mood modification* were observed. In other words, male gender and students with a low age were correlated with the presence of dependent and problematic players. In fact, students with a greater age, had more self control, while young students in comparison had a tendency to perceive more unpleasant emotions that occur when the game is suddenly interrupted or suspended, and thus, consequently, were more likely to experience irritability and mood swings [10].

The results of this study are in agreement with those of previous studies, where it is described that males are pathological players more so than females, and those of a younger age are more involved [6,7,10,35,46].

In addition, the variables *Daily gaming time* and *Daily game frequency* were positively correlated with all items of the GAS, except Item 4 (i.e., *Withdrawal*). In other words, the time dedicated to video games and the frequency of gaming represents predictors of probable dependence.

Finally, the *Education level* variable was negatively correlated with *Salience*, both in the univariate and the multivariate analyses, and negatively correlated only in the univariate analysis with *Relapse*, i.e., the *Education level* was only negatively correlated with some items.

By using logistic regression, a positive correlation between the *Monothetic GAS score* and *Polythetic Partial GAS* score with *Daily gaming time* was evidenced, in agreement with other studies [25,34,47]. This correlation was not present with the *Polythetic Global GAS score*; in this case, it was shown that *Daily game frequency* and *Education level* were a positive and negative predictor of *Polythetic Global GAS score*, respectively. We observed that when considering all items (including Items 1, 2, and 3), additional information was obtained, i.e., different predictors were individuated. In fact, *Daily game frequency* represents a clear symptom of game addiction [40] and is linked to gaming time [47,48], while a higher education level implicates a greater capacity of self-control and therefore less of a game addiction. This result is according tothe correlations between age and some items; in fact, generally, students with a high education level are of a greater age in comparison to students with a low education level according to Khazaal Y et al. (2016) [15]. Our results regarding time spent gaming and game addiction are in accordance with those of Griffiths M., et al., and Triberti S, et al. [40,48]; in fact, *Daily gaming time* is synonymous with dependence, as described by Brunborg G.S., et al. [32], but some authors argue that there is a need for more information to evaluate the impact considering additional activities or commitments of the subjects [40,49]. These results suggest that the dependence on video games linked to *Daily gaming time* is described by the *Polythetic Global GAS score*. In fact, a student who plays for five minutes but twenty times a day—i.e., for a total of 1 h and 40 min, shows behavior that indicates symptoms of dependence, expressed by the subject’s difficulty to break away from the video game. This information was individuated considering the *Polythetic Global GAS score*, while the *Polythetic Partial GAS score* did not individualize this correlation. Therefore, we observed that the use of the *Polythetic Partial GAS score* i.e., not considering Items 1, 2, and 3, implicates a loss of information and, in addition, the *Partial GAS* score furnishes results similarly to the *Monothetic GAS score*.

## 5. Conclusions

This study showed that, according to previous studies, males are pathological gamblers more so than females and spend more time playing video games. In particular, a high daily game frequency or daily gaming time is a symptom of video game addiction, while a higher education level, which generally corresponds to a greater age, is associated with a reduction in game addiction. Finally, the results obtained by using the *Polythetic* (*Global* and *Partial GAS*) and *Monothetic* structures suggest that analysis of the original *GAS (Polythetic Global GAS score)* should not be neglected.

In relation to the open question about the use of the GAS tool in the evaluation of video game addiction, these results should be further confirmed. Particularly, in a recent study on GAS validation, Khazaal Y et al., (2018) [50], suggested that the analysis of single items should also be considered. In addition, Lin, C. Y. et al. [51] tested the psychometric properties of the Persian GAS through both classical and modern test theories, showing that the Persian GAS is a reliable and valid instrument for healthcare providers to assess the level of gaming addiction among Persian-speaking adolescents.

In light of these recent studies, our results could contribute to increasing the knowledge on the use of the GAS, to offer a comparison with the results of other studies using this scale, and to enrich the ongoing debate regarding the evaluation of video game addiction.

### Limitations

In this study we did not distinguish between different types of video games, such as online and offline video games. We also did not evaluate other correlations such as the time spent studying or the reduction in primary or secondary school performance, as described by Brunborg, Mentzoni et al. [32]. In fact, it is possible that some games are likely correlated more to dependence, while other game types can stimulate study, i.e., the characteristics of a game can be of importance in the development of video game addiction [52]. Finally, to reduce the statistical bias connected to sample selection, the authors suggest a multicenter study. Regardless, these limitations do not diminish the statistical significance of the results.

## Figures and Tables

**Table 1:** Characteristics of the 622 participants in our study.

Parameters	Percentage/Mean ± SD	Statistical Analysis
*Age*	11.50 ± 1.128	—
*Gender*		
Male	52.89% (329/622)	52.89% > 47.11%, *p* = 0.161 (B)
Female	47.11% (293/622)	
*Education level*		
Elementary school	34.89% (217/622)	34.89% < 65.11%, *p* < 0.0001 * (B)
Secondary School	65.11% (405/622)	
*Day gaming time (hours)*		*p* < 0.0001 * (C)
One	12.38% (77/622)	Two hours, *p* < 0.0001 ** (Z)
Two	37.62% (234/622)	Three hours, *p* < 0.0001 ** (Z)
Three	37.78% (235/622)	One hours, *p* < 0.0001 *** (Z)
Four	12.54% (70/622)	Four hours, *p* < 0.0001 *** (Z)
≥Five	1.29% (8/622)	≥Five hours, *p* < 0.0001 *** (Z)
*Day gaming frequency*		
One	32.15% (200/622)	One hours, *p* < 0.0001 ** (Z)
Two	34.24% (213/622)	Two hours, *p* < 0.0001 ** (Z)
Three	32.48% (202/622)	Three hours, *p* < 0.0001 ** (Z)
≥Four	0.96% (6/622)	≥Four hours, *p* < 0.0001 *** (Z)
*Interview (pathologic*) +		*p* < 0.001 * (Q)
*Salience* = Item 1	41.96% (261/622)	Item 2, *p* < 0.0001 ** (MRD)
*Tolerance* = Item 2	64.15% (399/622)	Item 4, *p* < 0.0001 ** (MRD)
*Mood modification* = Item 3	20.26% (126/622)	Item 3, *p* < 0.0001 *** (MRD)
*Withdrawal* = Item 4	60.13% (374/622)	Item 7, *p* < 0.0001 *** (MRD)
*Relapse* = Item 5	53.22% (331/622)	
*Conflict* = Item 6	32.96% (205/622)	
*Problems* = Item 7	23.47% (146/622)	
*Interview GAS (pathologic*) ++		
*Monothetic GAS*	1.93% (12/622)	
*Polythetic Global GAS*	37.46% (233/622)	
*Polythetic Partial GAS*	4.50% (28/622)	

* = significant test; ** = most frequent; *** = less frequent; C = Multiple comparison χ^2^ test; Z = Z-test;B = Binomial test; Q = Cochran’s Q test; MRD= Minimum Required Differences method with Bonferroni p-value corrected for multiple comparisons; SD = standard deviation; + = pathological students, considering every Item; ++ = using Game Addiction Scale(GAS) scale of both Monothetic and Polythetic structure to define pathological students; *Monothetic Global GAS score =* we considered simultaneously all Items with a score ≥ 3; *Polythetic Global GAS score* = polythetic structure, including all Items and considering subjects with a minimum of 4 out of 7 Items with a score ≥ 3 as pathological; *Partial GAS score* = we considered simultaneously Items 4,5,6 and 7 with a score ≥ 3.

**Table 2:** Percentages of students’ answers for every items and every score considering a Likert scale of 1–5.

Interview +	Item 1	Item 2	Item 3	Item 4	Item 5	Item 6	Item 7
Score 1	41.96% (261/622)	16.72% (104/622)	69.77% (434/622)	23.31% (145/622)	29.74% (185/622)	50.32% (313/622)	61.90% (385/622)
Score 2	16.08% (100/622)	19.13% (119/622)	9.97% (62/622)	16.56% (103/622)	17.04% (106/622)	16.72% (104/622)	14.63% (91/622)
Score 3	28.94% (180/622)	40.84% (254/622)	10.78% (67/622)	31.20% (194/622)	31.20% (194/622)	19.29% (120/622)	14.47% (90/622)
Score 4	5.95% (37/622)	16.72% (104/622)	5.14% (32/622)	19.61% (122/622)	11.09% (69/622)	8.36% (52/622)	5.30% (33/622)
Score 5	7.07% (44/622)	6.59% (41/622)	4.34% (27/622)	9.32% (58/622)	10.93% (68/622)	5.31% (33/622)	3.70% (23/622)

+ = using a Likert scale of 1–5: 1 = never, 2 = rarely, 3 = sometimes, 4 = often, and 5 = very often.

**Table 3:** Univariate and multivariate linear correlation analyses between every item and independent variables: *Age*, *Gender, Education level, Daily gaming time (hours)*,and *Daily gaming frequency.*

Linear Correlation Analysis	Univariate Analysis *R* (*p*-Value)	Multivariate Analysis *(Rpartial*; *p*-Value)
		Multiple linear correlation coefficient = 0.476
*Salience* = Item 1/*Age*	−0.10 (0.0258) *	*Rpartial* = −0.03; *p* = 0.476
Item 1/*Gender*	−0.25 (< 0.0001) *	*Rpartial* = −0.15.; *p* = 0.0002 *
Item 1/*Education level*	−0.16 (0.0001) *	*Rpartial* = −0.10; *p* = 0.0178 *
Item 1/*Daily gaming time (hours)*	0.42 (<0.0001) *	*R partial* = 0.276; *p* < 0.0001 *
Item 1/*Daily gaming frequency*	0.31 (<0.0001) *	*Rpartial* = 0.166; *p* < 0.0001 *
		Multiple linear correlation coefficient = 0.434
*Tolerance* =Item 2/*Age*	0.03 (0.047)	*Rpartial* = 0.01; *p* = 0.843
Item 2/Gender	−0.22 (< 0.0001) *	*Rpartial* = −0.11; *p* = 0.0056 *
Item 2/*Education level*	0.01 (0.77)	*Rpartial* = 0.04; *p* = 0.318
Item 2/*Daily gaming time (hours)*	0.42 (< 0.0001) *	*Rpartial* = 0.32; *p* < 0.0001 *
Item 2/*Daily gaming frequency*	0.22 (< 0.0001) *	*Rpartial* = 0.07; *p* = 0.071
		Multiple linear correlation coefficient = 0.155
*Mood modification* = Item 3/*Age*	0.35 (0.39)	*Rpartial* = 0.04; *p* = 0.372
Item 3/*Gender*	0.05 (0.19)	*Rpartial*= 0.09; *p* = 0.0302 *
Item 3/*Education level*	0.01 (0.79)	*Rpartial*= −0.01; *p* = 0.88
Item 3/*Daily gaming time (hours)*	0.11 (0.007) *	*Rpartial*= 0.10; *p* = 0.0132 *
Item 3/*Daily gaming frequency*	0.09 (0.0257) *	*Rpartial* = 0.05; *p* = 0.219
		Multiple linear correlation coefficient = 0.093
*Withdrawal* =Item 4/*Age*	0.003 (0.93)	*Rpartial* = −0.03; *p* = 0.53
Item 4/*Gender*	−0.07 (0.09)	*Rpartial* = −0.05; *p* = 0.21
Item 4/*Education level*	0.03 (0.51)	*Rpartial* = 0.04; *p* = 0.30
Item 4/*Daily gaming time (hours)*	0.07 (0.10)	*Rpartial*= 0.04; *p* = 0.32
Item 4/*Daily gaming frequency*	0.03 (0.41)	*R partial* = 0.01; *p* = 0.72
		Multiple linear correlation coefficient = 0.381
*Relapse* = Item 5/*Age*	−0.11 (0.007) *	*Rpartial* = −0.05; *p* = 0.22
Item 5/*Gender*	−0.11 (0.005) *	*Rpartial* = −0.01; *p* = 0.72
Item 5/*Education level*	−0.12 (0.003) *	*Rpartial* = −0.01; *p* = 0.77
Item 5/*Daily gaming time (hours)*	0.34 (< 0.0001) *	*Rpartial* = 0.25; *p* < 0.0001 *
Item 5/*Daily gaming frequency*	0.28 (< 0.0001) *	*Rpartial* = 0.14; *p* = 0.0005 *
		Multiple linear correlation coefficient = 0.32
*Conflict* = Item 6/*Age*	0.02 (0.59)	*Rpartial* = 0.06; *p* = 0.17
Item 6/*Gender*	−0.02 (0.61)	*Rpartial* = 0.07; *p* = 0.07
Item 6/*Education level*	−0.03 (0.39)	*Rpartial* = −0.04; *p* = 0.34
Item 6/*Daily gaming time (hours)*	0.29 (< 0.0001) *	*Rpartial* = 0.25; *p* < 0.0001 *
Item 6/*Daily gaming frequency*	0.19 (< 0.0001) *	*Rpartial* = 0.07; *p* = 0.072
		Multiple linear correlation coefficient = 0.336
*Problems* = Item 7/*Age*	0.09 (0.0491) *	*Rpartial* = 0.11; *p* = 0.005 *
Item 7/*Gender*	−0.09 (0.033) *	*Rpartial* = −0.001; *p* = 0.98
Item 7/*Education level*	−0.002 (0.95)	*Rpartial* = −0.05; *p* = 0.22
Item 7/*Daily gaming time (hours)*	0.30 (< 0.0001) *	*Rpartial* = 0.21; *p* < 0.0001 *
Item 7/*Daily gaming frequency*	0.22 (< 0.0001) *	*Rpartial* = 0.12; *p* = 0.002 *

* = significant test; R = Pearson’s linear correlation coefficient; *R_partial*= the partial correlation coefficient is the coefficient of correlation of the variable with the dependent variable, adjusted for the effect of the other variables in the mode.

**Table 4:** Logistic regression between Monothetic GAS score, Polythetic Global GAS score, Polythetic Partial GAS score, and the independent variables (i.e., Age, Gender, Education level, Daily gaming time (hours), and Daily gaming frequency).

Logistic Regression	Coefficient	Standard Error	OR	95% CI	*p*-Value
Null model vs. full model					0.0071 (C)
*Monothetic Global GAS*/*Age*	0.07	0.34	1.07	0.56–2.08	0.83
*Monothetic Global GAS/Gender*	−0.01	0.64	0.99	0.28–3.48	0.99
*Monothetic Global GAS/Education level*	−0.02	0.94	0.98	0.16–6.16	0.98
*Monothetic Global GAS/Daily gaming time (hours)*	1.31	0.47	3.70	1.46–9.36	0.0057 *
*Monothetic Global GAS/Daily gaming frequency*	0.44	0.45	1.56	0.64–3.78	0.33
Constant	–9.66	3.40			0.0046 *
Null model vs. full model					<0.0001 (C)
*Polythetic Global GAS/Age*	0.16	0.11	1.18	0.95–1.45	0.13
*Polythetic Global GAS/Gender*	0.18	0.19	0.84	0.57–1.22	0.35
*Polythetic Global GAS/Education level*	−0.42	0.29	2.64	2.03–3.42	<0.0001 *
*Polythetic Global GAS/Daily gaming time (hours)*	0.97	0.13	0.66	0.38–1.16	0.15
*Polythetic Global GAS/Daily gaming frequency*	0.34	0.12	1.40	1.10–1.78	0.0063 *
Constant	−4.82	1.03	—	—	<0.0001 *
Null model vs. full model					0.0011 (C)
*Polythetic Partial GAS/Age*	0.08	0.23	1.08	0.69–1.70	0.73
*Polythetic Partial GAS/Gender*	0.40	0.41	1.49	0.66–3.34	0.33
*Polythetic Partial GAS/Education level*	−0.18	0.63	0.83	0.24–2.84	0.77
*Polythetic Partial GAS/Daily gaming time (hours)*	0.77	0.28	2.16	1.25–3.72	0.0059 *
*Polythetic Partial GAS/Daily gaming frequency*	0.55	0.29	1.74	0.98–3.10	0.06
Constant	−7.26	2.25			0.0012 *

* = significant test; OR = odds ratios; CI = odds ratios confidence interval at 95%; null model= −2ln(L_0_), where L_0_ is the likelihood of obtaining the observations if the independent variables do not affect the outcome, the full model: −2ln(L_0_), where L_0_ is the likelihood of obtaining the observations with all independent variables incorporated in the model; C = chi-square test.
